# DTIPrep: quality control of diffusion-weighted images

**DOI:** 10.3389/fninf.2014.00004

**Published:** 2014-01-30

**Authors:** Ipek Oguz, Mahshid Farzinfar, Joy Matsui, Francois Budin, Zhexing Liu, Guido Gerig, Hans J. Johnson, Martin Styner

**Affiliations:** ^1^Department of Electrical and Computer Engineering, University of IowaIowa City, IA, USA; ^2^Department of Psychiatry, University of North Carolina at Chapel HillChapel Hill, NC, USA; ^3^Department of Psychiatry, University of IowaIowa City, IA, USA; ^4^School of Biomedical Engineering, Southern Medical UniversityGuangzhou, China; ^5^SCI Institute, University of UtahSalt Lake City, UT, USA

**Keywords:** diffusion MRI, diffusion tensor imaging, quality control, software, open-source, preprocessing

## Abstract

In the last decade, diffusion MRI (dMRI) studies of the human and animal brain have been used to investigate a multitude of pathologies and drug-related effects in neuroscience research. Study after study identifies white matter (WM) degeneration as a crucial biomarker for all these diseases. The tool of choice for studying WM is dMRI. However, dMRI has inherently low signal-to-noise ratio and its acquisition requires a relatively long scan time; in fact, the high loads required occasionally stress scanner hardware past the point of physical failure. As a result, many types of artifacts implicate the quality of diffusion imagery. Using these complex scans containing artifacts without quality control (QC) can result in considerable error and bias in the subsequent analysis, negatively affecting the results of research studies using them. However, dMRI QC remains an under-recognized issue in the dMRI community as there are no user-friendly tools commonly available to comprehensively address the issue of dMRI QC. As a result, current dMRI studies often perform a poor job at dMRI QC. Thorough QC of dMRI will reduce measurement noise and improve reproducibility, and sensitivity in neuroimaging studies; this will allow researchers to more fully exploit the power of the dMRI technique and will ultimately advance neuroscience. Therefore, in this manuscript, we present our open-source software, DTIPrep, as a unified, user friendly platform for thorough QC of dMRI data. These include artifacts caused by eddy-currents, head motion, bed vibration and pulsation, venetian blind artifacts, as well as slice-wise and gradient-wise intensity inconsistencies. This paper summarizes a basic set of features of DTIPrep described earlier and focuses on newly added capabilities related to directional artifacts and bias analysis.

## 1. Introduction

Thousands of diffusion MRI (dMRI) datasets are collected every day across the world in studies of autism (Wolff et al., 2012), schizophrenia (Gilmore et al., 2010), Huntington's disease (Dumas et al., 2012), Alzheimer's disease (Rose et al., 2000), Parkinson's disease, substance abuse (Parnell et al., 2009; Coleman et al., 2012) and many other conditions. White matter (WM) degeneration is often identified as a crucial biomarker for all these diseases. The tool of choice for studying WM is diffusion MR imagery. dMRI imaging extends the capabilities of conventional MRI methods by measuring the diffusion properties of the tissue. Several investigators have proposed dMRI approaches to depict WM maturation (Basser and Pierpaoli, 1998; Shrager and Basser, 1998; Barkovich, 2000; Geng et al., 2012), and researchers (Zhang et al., 2002, 2005) have shown that measurements extracted from dMRI, such as Fractional Anisotropy (FA) and Mean Diffusivity (MD), are more stable than absolute MRI intensity measures. As a result, extensive research efforts have utilized dMRI in both normal subjects and patients in an attempt to yield new insights into the microstructural organization of WM that are not available with conventional MRI (Rose et al., 2000; Ciccarelli et al., 2001; McKinstry et al., 2002). Additionally, dMRI provides a wealth of information for assessing not only the volume and morphology of specific brain regions, but also the organization of these regions, and allows the description of fiber tracts within the brain. Tractography techniques (Mori and Zijl, 2002), which estimate paths of brain WM fiber bundles based on dMRI data, can identify abnormalities in fiber shape or microstructure along the fiber bundles (Escolar et al., 2009). Connectivity studies (Hagmann et al., 2008; Boucharin et al., 2011; Oguz et al., 2012a) can further elucidate pathologies by analyzing the strength of connections between distant regions of the brain. dMRI can be applied both in the clinical setting and in pre-clinical animal research, as the dMRI data can be acquired and processed using similar methodology in humans and animals (Gerig et al., 2011; Oguz et al., 2012b). Thus, findings have the potential to directly translate from basic to clinical science.

**Figure 1:**
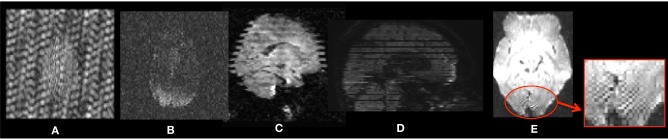
**Examples of intensity artifacts detected with DTIPrep**. **(A)** An electromagnetic interference-like artifact, **(B)** severe signal loss in the anterior and middle regions, **(C)** venetian blind artifact, **(D)** inter-slice and intra-slice intensity artifact, and **(E)** checkerboard artifact.

**Figure 2:**
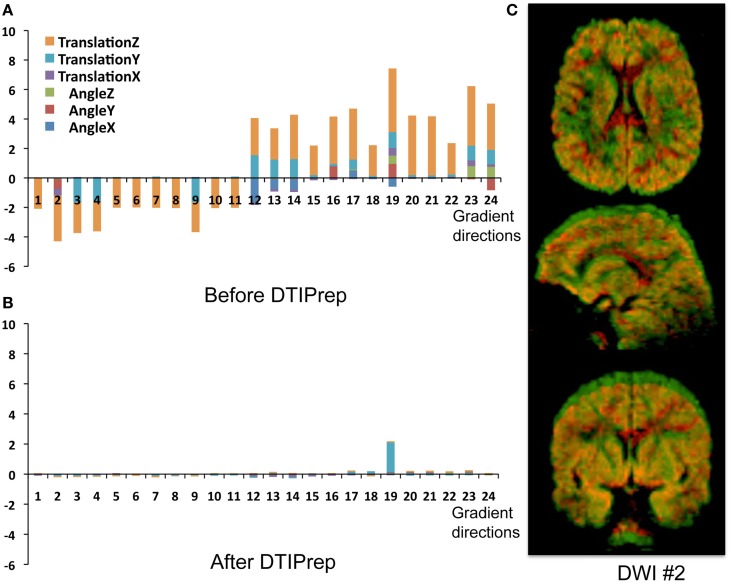
**Head-motion artifacts**. Rigid registration parameters between each gradient and baseline image of the original DWIs **(A)** and corrected DWIs **(B)**. **(C)** Overlay comparison between original DWI #2 before (red) and after (green) correction.

dMRI technology has enormous potential, but it suffers from a unique and complex set of image quality problems, limiting the sensitivity of dMRI studies and reducing the accuracy of findings. dMRI data is obtained by acquiring a series of images using non-collinear diffusion-sensitizing gradients (called DWI, Diffusion Weighted Images) and fitting a mathematical model at each image location to this data, e.g., a tensor in the case of Diffusion Tensor Imaging (DTI). The acquisition time for dMRI is longer than conventional MRI due to the need for multiple DWI acquisitions. An increased acquisition time allows for more motion artifacts, reduced signal-to-noise ratio (SNR), and increased proneness to a wide variety of artifacts, including eddy-current and motion artifacts, “venetian blind” artifacts, as well as slice-wise and gradient-wise inconsistencies (Figures 1, 2). In addition, dMRI is a mechanically demanding scan: the acquisition protocols are inherently complex, often pushing the mechanical limits of scanners due to fast gradient switching in echo-planar imaging. Many artifact types plague diffusion imagery, and many of these are unavoidable even with the most conservative acquisition protocols. Therefore, unlike conventional MRI data, dMRI data needs to be carefully checked and corrected to remove these artifacts, making a quality control (QC) procedure absolutely necessary for dMRI studies. Blindly using these complex scans may bias data collection and thus affect the results of research studies (Liu et al., 2010; Lauzon et al., 2012).

**Figure 3:**
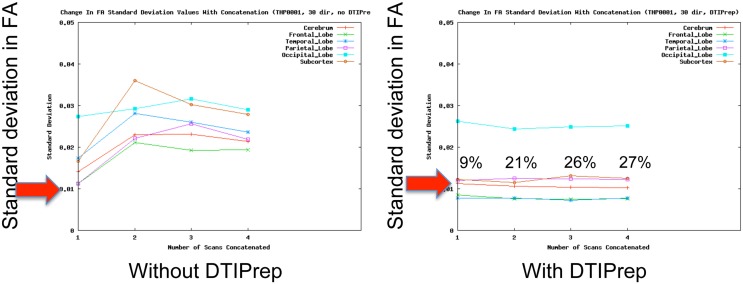
**QC reduces measurement noise in DTI**. Standard deviation of FA in five healthy subjects in six regions are shown. QC reduces FA standard deviation considerably even for a single scan; the improvement is dramatic when the number of included repeat scans (horizontal axis) is increased. Percentage figures indicate number of gradient directions excluded by DTIPrep. Courtesy of V. Magnotta (University of Iowa).

QC of dMRI data is a critical issue for the successful use of this technique in studies, and tools for adequately addressing this matter are crucially needed by the medical imaging community. Thorough QC of dMRI will lead to increased sensitivity in neuroimaging studies, which will help advance neuroscience and improve our understanding of many disease processes as well as of the healthy maturation process of the brain. Undetected, dMRI artifacts result in severely increased measurement noise in the data and even may result in confounding factors leading to wrong conclusions. This is well illustrated by a quantitative study of data from the PredictHD project (focused on Huntington's disease) using DTIPrep to QC images from five normal controls at eight different scanning sites (30 gradient directions for each scan). The standard deviation of Fractional Anisotropy (FA) before and after QC for six different regions of interest is shown in Figure 3. The standard deviation of FA is decreased even in a single scan and the improvement becomes dramatic when multiple scans are included. The increased measurement noise leads to loss of sensitivity in studies. Furthermore, affected tensors (or equivalent diffusion models) can also disrupt tractography methods and result in errors in fiber orientation or premature fiber termination (Figures 6B,C).

### 1.1. Illustrative case study

QC statistics from an ongoing multi-site DTI study in autism best illustrate the necessity of DTI QC: of over 400 datasets, 14% of the datasets were fully rejected by the QC (due to scanning issues, motion and high noise level); all other datasets were corrected such that about 20% of these would have been considered unusable without correction and about 50% showed considerable gain in signal. This example well illustrates that application of DTI QC and data correction also brings a significant economical benefit by saving scans which otherwise would need to be eliminated from the study. This is even more important in longitudinal studies where missing data prevents calculation of individual temporal trajectories.

Unfortunately, the need for rigorous QC is a severely under-recognized issue in the dMRI community. Most current studies are extremely limited in terms of QC and they commonly only perform correction for motion artifacts and eddy-currents [typical examples: Li et al. (2010), Pannek et al. (2011), Yan et al. (2011), Zhang et al. (2012)]. Such studies may therefore be prone to decreased sensitivity and accuracy. In a quantitative study, DTIPrep was used to QC data from five normal controls at different scanning sites (30 gradient directions for each scan). The standard deviation of FA before and after QC for six different regions of interest is shown in Figure 3. The standard deviation of FA is decreased even in a single scan, as represented by the first data point in each graph; this is indicative of decreased measurement noise, which leads to increased sensitivity in studies. The improvement becomes dramatic when multiple scans are included; the flat curves in the after-QC graph indicate highly reproducible measurements become possible across sites when DTIPrep is used for QC, in contrast to the highly variable data points in the before-QC graph.

We recognize that some of the functionality in DTIPrep has already been published and is available to the community. These include Tortoise^1^ (Pierpaoli et al., 2010), which is a software package for processing dMRI data and consists of two main modules, one for preprocessing and one for analysis, including tensor fitting and ROI analysis. The preprocessing stage for Tortoise currently includes utilities for motion correction and eddy-current correction. Similarly, the FSL platform offers a diffusion toolbox, FDT, which includes both command line tools and a user interface for common DTI analysis tasks such as tensor estimation, registration, tractography and computation of probabilistic connectivity maps (Behrens et al., 2007; Woolrich et al., 2009). Current versions of FDT provide QC support mostly limited to eddy current correction and motion correction. Additionally, JIST (Lucas et al., 2010) is another popular image processing environment, including utilities for analysis of DWI such as model fitting, visualization and tractography. JIST and the related MIPAV platform are designed to allow a wide variety of plugins to be developed by external users which include general-purpose (not dMRI-specific) QC methods. Similarly, AFNI (Cox and Hyde, 1997) and SPM (Friston et al., 2007) are commonly used software packages that include general-purpose pre-processing functionality. DTIPrep has functionality that significantly expands upon these preprocessing steps, as discussed in section 2.

Additionally, most of these are research-oriented pieces of software that are often not generally applicable to data from multi-site, multi-scanner studies with non-uniform data formats, acquisition protocols and more. The target audience for DTIPrep is physicians and neuroscientists. Through extended documentation and a streamlined user-friendly interface, DTIPrep aims to fill a very important gap in the toolkits of the users in the dMRI community. One simple example of this level of abstraction provided to the users is the data input mechanism to DTIPrep, which was recently revamped to support 42 different variants of the DICOM format, which required a massive software development effort. In practice, this means the physician can now load their data into DTIPrep without having to worry about the particular brand, model or software version of their scanner, and through the automated pipeline in DTIPrep, quickly create a clean version of their data that is readily exportable in many industry-standard formats. Additionally, DTIPrep can also enforce protocol compliance, which is non-trivial in the large multi-site, multi-scanner studies, and it can catalog identified artifacts such that meta-analysis becomes possible over the scope of the entire study.

In this paper, we present DTIPrep, a unified, easy-to-deploy platform that addresses data quality problems that affect dMRI, and demonstrate its effectiveness on real data. An overview of the software and algorithms are provided, with a focus on the two most recently added features, targeting directional artifacts and bias analysis.

## 2. Materials and methods

**Figure 4:**
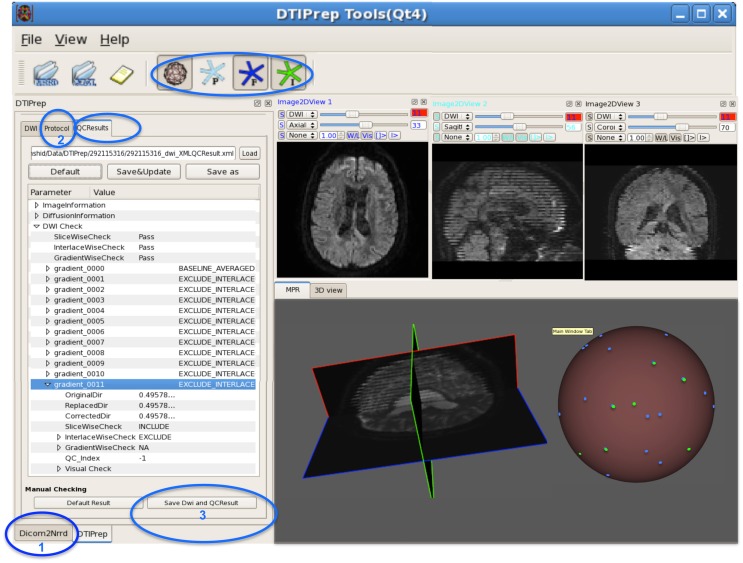
**DWI-based QC results using DTIPrep through three steps**. **(1)** converting the DWI image from DICOM to NRRD format, **(2)** loading the protocol and running the software, and **(3)** if necessary, visual checking and saving the final DWI dataset. In this example, gradient #11 suffers from intensity artifact and is excluded. The sphere shows a 3D view of the gradient distribution before (blue dots) and after running DTIPrep (green dots, visualized on top of the blue dots), respectively. In this particular example, a large number of DWI's were excluded (missing green dots on the 3D sphere). The 3D sphere also reveals a highly non-uniform distribution of the input diffusion gradient, indicating a non-optimal acquisition protocol.

DTIPrep is a dedicated dMRI QC software platform developed to identify and correct all common, known dMRI artifacts, with a special focus on handling data from multi-site studies. DTIPrep is fully open-source (BSD License) and publicly available on the NITRC website^2^. The implementation uses the open-source ITK and VTK toolkits and the graphical user interface is built with Qt4. Figure 4 illustrates the GUI and highlights the high-level workflow in DTIPrep. The QC process is separated into two phases: (1) a fully automatic phase for quality assessment and artifact correction/removal, and (2) a visual assessment phase for both the DWI volumes and the reconstructed DTI data. In the automatic phase, all datasets are changed (minimally via the eddy-current and motion correction step, if enabled); in contrast, only a minority of cases need correction in the visual assessment stage (in the autism dataset described above as case study, only 5% of the data required manual intervention).

The automatic QC phase is controlled by a study-specific protocol file that stores all QC parameters. Each individual step (described below) can be enabled/disabled and adapted via parameter settings using this protocol file. A detailed XML-based report of each QC metric is written for *post hoc* meta-analysis. The meta-analysis of the QC reports has proven to be a valuable tool in identifying subtle site- or scanner-specific MR hardware anomalies that can be dynamically addressed to ensure the highest quality data collection.

**Figure 5:**
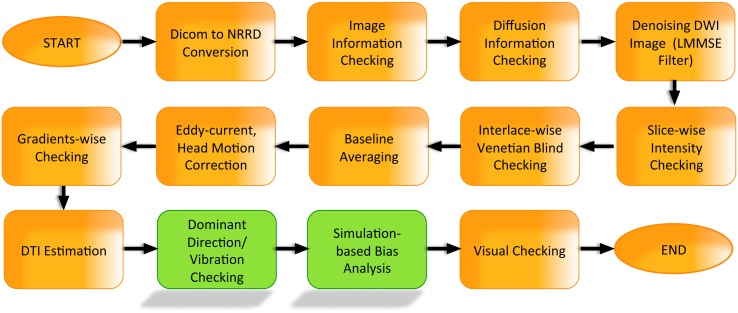
**DTIPrep workflow**.

More specifically, the automatic QC phase performs the following steps (Liu et al., 2010) (Figure 5):

1. DICOM to NRRD conversion (NRRD is a popular file format for dMRI data, capable of storing all diffusion information within an ASCII readable file header).
2. Image information checks (ensuring correct image dimensions, spacing, and orientation).
3. Diffusion information checks (ensuring correct diffusion gradient orientations, gradient *b*-values).
4. Rician noise removal on raw DWI volumes (Tristán-Vega and Aja-Fernández, 2010).
5. Inter-slice brightness artifact detection via normalized correlation analysis between successive slices within a single DWI volume.
6. Interlaced correlation analysis for detection and removal of “venetian blind” artifacts and motion within a single DWI volume.
7. Co-registration to an iterative average over all the baseline images.
8. Eddy-current and motion artifact correction, including appropriate gradient direction adjustments.
9. Residual motion detection to ensure all DWI volumes are well registered.
10. Reconstruction of the DTI data and computation of DTI property maps. Currently, DTIPrep implements a single tensor model via weighted least-squares fit (Goodlett et al., 2007) followed by computing the standard tensor property maps, which include fractional anisotropy (FA) (Basser and Pierpaoli, 1995), mean diffusivity (MD) (Zhai et al., 2003), axial diffusivity (AD) and radial diffusivity (RD) (Pierpaoli and Basser, 1996).
11. Directional artifact detection/correction. This step is a recent algorithmic development and will be discussed in more detail below in section 2.1.

Note that all the algorithms currently in DTIPrep apply equally to single-tensor analysis (DTI) and to higher-order models, with the exception of the final steps of model estimation and directional artifact detection. Further, these techniques can accommodate single or multiple *b*-value acquisitions.

DWI volumes found to be affected by corrupting artifacts (steps 5, 6, 9) are removed from the DWI scan. While some of these artifacts may be local and not affect the entire volume, local removal would lead to different regions of the brain having quite different signal to noise (SNR) values due to the varying number of DWI's locally available. Data with such a varying SNR would complicate subsequent analysis to avoid bias. The gain achieved from local rather than global rejection of affected DWI's is not significant enough to justify the added level of complexity. Thus, we have made a design decision to fully reject the DWI's along affected gradient directions. Local approaches also exist, such as the RESTORE method (Chang et al., 2005) which locally identifies potential outliers and rejects them. It should also be noted that even with the rejection of entire DWI volumes, the different noise patterns in the scans may cause inter-subject SNR and bias differences. DTIPrep outputs the included/excluded DWI directions in its automatically generated QC report for subsequent statistical analysis.

After steps 5, 6, and 9, DTIPrep further checks whether enough DWI's have been retained. The threshold ratio below which the whole dataset is rejected is a parameter adjustable in the protocol, commonly set between 30% and 40% of the full set of DWI's.

After the automatic QC phase, a visual assessment step is recommended. In this visual assessment phase, first, all DWI volumes are efficiently checked within DTIPrep to detect and remove remaining DWI data with artifacts. Additional remaining artifacts happen rarely in our experience (e.g., in the autism dataset described above as our case study, only 5% of the data required manual intervention); but it is still a necessary step to ensure proper QC. Finally, the reconstructed DTI image is assessed within 3D Slicer^3^, via a visualization of the local tensor orientations as well as investigative, interactive fiber tractography to assess anatomical fiber correctness. Errors in local orientations are corrected by editing the DWI gradient measurement frame. Abnormal fiber tractography indicates either severe WM pathology or a scanning issue necessitating the rejection of this dataset. A detailed user manual for this step is publicly available on DTIPrep's NITRC page.

### 2.1. Automatic detection and correction of directional artifacts

Recent studies [Hiltunen et al., 2006, Gallichan et al., 2010, Farzinfar et al., 2012, 2013b] have demonstrated a new kind of artifact that was not previously detected by DTIPrep. These artifacts manifest themselves as a strong bias in the measured principal direction of diffusion (PD). They are sometimes accompanied by pronounced local signal loss in the diffusion-weighted images. It is believed that these artifacts are caused by the vibrations of the scanner table during the scan, which can have a non-negligible effect especially in subjects under 30 kg (Liu et al., 2011). The detection and correction/removal of these artifacts is therefore especially crucial for pediatric studies as well as primate and rodent studies. In our experience, this type of artifact can occur in up to 50% of scans in pediatric studies in an affected scanner. It is noteworthy that newer scanner models (installed after 2011) show a significantly reduced occurrence rate of such artifacts (less than 5% scans).

**Figure 6:**
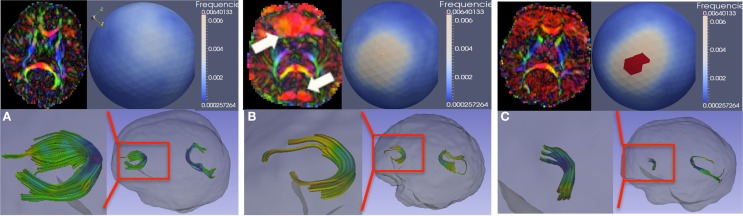
**Vibration artifacts**. **(A)** Artifact free scan. Top-left, a representative axial slice of the color-FA map. Top-right, spherical histogram of the PD distribution within the entire brain. Bottom-right, tractography of genu and splenium of the corpus callosum. Bottom-left, genu tract in more detail. **(B)** Vibration artifacts may manifest as localized (prefrontal region for this example) signal-loss in the DWI image or as dominant L-R (red) direction. **(C)** Vibration artifact in the absence of localize DWI signal loss. Spherical viewpoints chosen to show locations of highest histogram frequency.

Figure 6 demonstrates such artifacts, both with (Figure 6B) and without (Figure 6C) localized signal loss. Figure 6A shows an artifact-free scan for comparison. These artifacts can be detected visually by identifying either a local dominant “color” or a widespread dominant “color” in the color-coded FA image, where the color denotes the PD (red: left-right, green: posterior-anterior, blue: superior-inferior). This is illustrated as the red (left-right) artifact observed locally in the frontal and posterior lobes in Figure 6B and more diffusely globally in Figure 6C. The spherical distribution of the PD displays no clustering for the artifact-free scan, whereas the dominating direction artifact generally prompts a higher degree of clustering in the spherical PD distribution, as shown with a mid-level clustering in Figure 6B and high-level in Figure 6C. The figure also illustrates the tractography of the anterior (genu) and posterior (splenium) fibers of the corpus callosum in each scan; clearly, the tractography is considerably affected in cases with artifacts.

Traditional artifact detection algorithms are designed to detect abrupt intensity changes in one slice or along one gradient direction. However, the vibration artifact presents itself either as a global disruption in the intensities or as local, gradual intensity change in neighboring slices over a subset of DWI's. To detect these artifacts, we have recently proposed a novel approach using an entropy-based measurement of the PD distribution (Farzinfar et al., 2012, 2013b). The orientation distribution is computed via a spherical histogram, using an icosahedron subdivision scheme to create the histogram bins. The entropy of this distribution quantifies the scattering and spread of the PD and is invariant to the patient's position in the scanner. High entropy indicates that the PD are distributed relatively uniformly. Low entropy demonstrates clustering of the PD distribution. Significant global clustering, as in Figures 6B,C (upper right, spherical distribution), indicates the presence of an artifact of dominating diffusion direction. Based on this orientation-based QC, dominant direction artifacts can be successfully detected and corrected.

#### 2.1.1. Detection stage

The proposed method (Farzinfar et al., 2012, 2013b) evaluates the entropy of the orientation distribution within WM, gray matter and whole brain regions. In order to detect scans with suspicious values of entropy (lower values of entropy for artifacts of dominating directions), we compare a given DT scan's entropy to values learned from a training set of artifact-free samples. Note that this algorithm takes advantage of the “default” pattern of directional distribution in the brain: given the anatomy of the WM, every brain is expected to have certain amounts of locations with predominantly left-right direction of diffusion (e.g., corpus callosum, anterior commissure), predominantly anterior-posterior direction of diffusion (e.g., inferior and superior fasciculi), and predominantly inferior-superior direction of diffusion (e.g., brain stem and internal capsule). The orientation distribution computed from the DTI is expected to reflect this underlying anatomy rather than being uniformly distributed on the unit sphere. The training set is used to compute estimates for this expected distribution of orientation in a given population. These trained entropy values are relatively sensitive to certain study settings; in particular, the WM maturation level and the voxel resolution relative to the brain size directly influences the underlying orientation distribution and therefore the entropy. This means that populations with considerably different brain sizes and maturation (e.g., neonate vs. 1 year old vs. adult) would need separately trained entropy settings. Given the expected value of entropy and its distribution for the population, we use *z*-scores to categorize the quality of a DT scan into three categories: acceptable (*z* < 1.64 Ð 90th percentile), suspicious (*z* ≥ 1.64 Ð 90th percentile), and unacceptable (*z* ≥ 2.58 Ð 99th percentile).

#### 2.1.2. Correction stage

We have proposed (Farzinfar et al., 2012, 2013b) an iterative leave-one-out-strategy over all individual DWI images by re-computing the DTI and the corresponding entropies. The DWI whose omission yields the most improvement in entropy is excluded from the scan. This process is repeated until the *z*-score is in acceptable range. However, for scans showing significant dominating direction artifacts, the whole scan is rejected. Our experiments have shown that if there is a strong directional bias, the gradient directions chosen for removal are not uniformly distributed on the unit sphere, but rather, cluster along a single plane. While removing the DWI along these directions may lead to an acceptable entropy level, the non-uniform distribution of the remaining gradient direction leads to a strong bias (as discussed below in section 2.2), ultimately rendering the image unsuitable for analysis. Therefore, we only apply the correction stage to the scans with low to moderate levels of these artifacts.

### 2.2. Simulation-based bias analysis

In addition to the artifact detection and correction steps discussed so far, we are currently integrating a bias analysis module to DTIPrep. A Monte Carlo (MC) simulation technique can be used in order to assess the bias and expected error for a given acquisition scheme. These simulations allow the user to predict the propagation of error in subsequent analysis and to understand bias in the estimated diffusion model. Clearly, an increase in error creates a need to increase the number of subjects to maintain the same level of sensitivity to group differences in neuroimaging studies. The bias in the diffusion estimation is caused by the acquisition noise, which follows a Rician distribution. At the typically low range of SNR of dMRI scans, this directionally-dependent noise distribution can cause a considerable bias in the diffusion model estimation if the gradient direction distribution is non-uniform.

**Figure 7:**
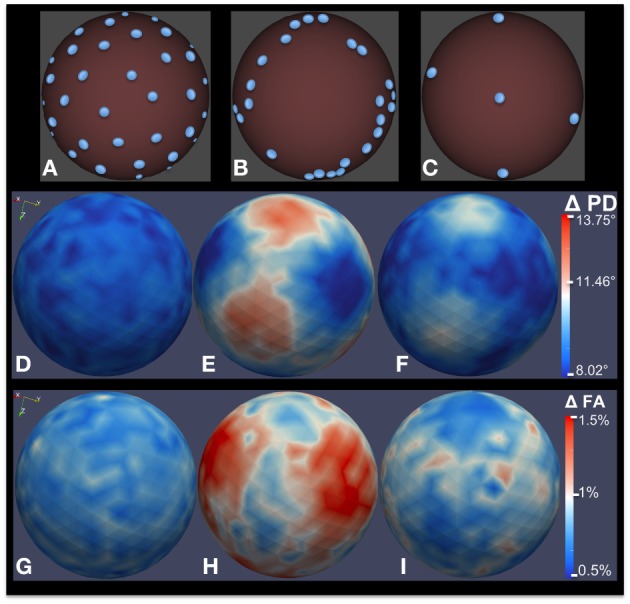
**Bias analysis of different DWI schemes via MC simulation**. **(A–C)** Gradient direction distribution for three common DWI acquisition schemes: 42-direction quasi-uniform, Phillips 32-direction non-uniform, and 6-direction uniform. **(D–F)** Estimated error distribution in PD computation. **(G–I)** Estimated error distribution in FA computation as a percentage of true FA. 200,000 MC simulation were performed for this experiment, with a true FA value of 0.4 and SNR = 10.

Many studies have investigated the optimal number and distribution of gradient sampling schemes for DWI acquisition protocols, based on Monte Carlo simulations (Jones et al., 1999; Jones, 2004), with the goal of minimizing measurement bias in estimated diffusion properties including fractional anisotropy (FA), mean diffusivity (MD), and the principal direction of diffusion (PD). In Farzinfar et al. (2013a), we proposed a technique similar to the one presented in Jones (2004) for bias estimation. Given a gradient sampling scheme, the *b*-value, and the true diffusion tensor, the MC simulation starts by computing the true signal intensities for the DWI. In each iteration, first, the true diffusion tensor is rotated randomly. Then, given the SNR level of choice, an appropriate Rician noise is added to the signal intensity along each gradient direction. These noisy signals are then used to compute the “measured” tensor of diffusion. The measurement error is computed as the difference between the true tensor and this noisy simulated tensor. Similarly, diffusion parameters such as FA and PD are computed from the noisy tensor and compared to their true values. This process is repeated hundreds of thousands of times to obtain a robust estimate of error. This approach also allows us to evaluate multiple runs and varying *b*-values. Note that a similar approach can be used for higher order diffusion representations modeling multiple tracts via standard tensor or fiber models. Furthermore, it is possible to visualize the estimated distribution of the error using DTIPrep (Figure 7).

This advanced functionality provides guidance for acquisition protocol selection. MC simulations allow the DTI-based analysis studies to select the best gradient sampling scheme for image acquisition for robust estimation of anisotropy and PD orientation. We are currently integrating this functionality into DTIPrep to make it readily available to the dMRI community.

## 3. Results

### 3.1. Directional artifacts

In the illustrative studies mentioned before, pediatric subjects were scanned at four different collaborating sites (Wolff et al., 2012) using Siemens Tim Trio 3T scanners (Hazlett et al., 2012). In that study, we have found the vibration artifact to be a serious and widespread problem. After discovery of the artifacts at all four sites, a hardware fix that Siemens has recently devised (Liu et al., 2011) has been adopted. For these four sites, before the hardware fix, the occurrence rate of the vibration artifacts were 1%, 5%, 54%, and 12%, respectively, in 6 months old subjects (*n* = 182). Following the hardware fix, the occurrence rate was reduced to less than 5% in the first three sites, but remained at 9% for the last site. This demonstrates the complexity of the provenance of these directional dominance artifacts. Clearly, not using the hardware fix is a serious problem that can lead to artifacts in more than half of the scans at some scanning sites. However, the data also demonstrates that the hardware fix is not a cure-all and there are studies that have considerably high artifact occurrence rates even with the use of the hardware fix. This demonstrates the necessity of thorough QC using post-acquisition software to avoid introducing severe bias in neuroimaging studies. Note that the hardware fix and the software solutions proposed here are completely unrelated approaches. We have applied the proposed entropy-based detection scheme to the scans and compared the findings to the visual assessment by a human expert. We were able to automatically detect 100% of the severe and 67% of the milder vibration artifacts.

### 3.2. Bias analysis

Figures 7A–C shows three different gradient sampling schemes with different levels of uniformity: a quasi-uniform distribution with 42 gradients based on electrostatic repulsion (Jones et al., 1999), a non-uniform distribution with 32 gradients that is standard on Philips scanners, and a uniform six gradient distribution. The MC simulation method is used to assess the bias in FA and PD estimates from data acquired with each protocol. The figure illustrates bias given a true tensor with an FA value of 0.4 and SNR = 10. 200,000 iterations were used in the MC simulation. The estimated error distributions in the PD (Figures 7D–F) and FA (Figures 7G–I) are shown. Note that, although the number of gradients in the non-uniform 32-gradient scheme is larger than the uniform six-gradient scheme (at the same overall SNR), the error in the PD and FA is considerably larger. For both tractography and anisotropy studies, the quasi-uniform 42-scheme would be the best scheme and the non-uniform 32-scheme would be the worst among the compared schemes.

These results illustrate an intuitive fact that has been known for over a decade (Jones et al., 1999): non-uniform gradient sampling schemes introduce strong biases. However, we still see clinical studies published in prestigious journals using highly non-uniform and thus suboptimal acquisition protocols, obviously unaware of eventual limitations in sensitivity and eventual confounding biases related to the choice of the dMRI sequence. This highlights two important conclusions: (1) Raising awareness of bias problems in dMRI studies is of crucial importance. (2) Whether studies start out with quasi-uniform or non-uniform acquisition protocols, it is important to assess the estimated bias for each subject after motion correction and artifact removal.

## 4. Discussion

DTIPrep offers a publicly available, unified platform to address many quality issues in dMRI datasets. We aim to continue to developing DTIPrep to expand its functionality. In particular, the two recent additions to DTIPrep, the directional artifact detection/correction and the simulation-based bias analysis, are both under active development as discussed below.

In addition to the proposed detection-and-removal approach for the directional artifacts discussed in section 2.1, we are also currently experimenting with alternative approaches to deal with directional artifacts. As previously discussed, the main approach we propose consists of detecting global abnormalities in the PD distribution, and removing these from the data. As alternative approaches, we are investigating (1) detecting local abnormalities and removing such artifacts; and (2) instead of removing the biased data, including it in the model estimation, with an explicit term to account for the bias.

1. Local detection/removal of artifacts: The approach currently implemented in DTIPrep assumes that a directional artifact will either affect the whole image or at least a large portion of the brain, which is necessary for the global entropy to be affected. However, in our experience, some scans will show only very localized signal loss which leads to directional artifacts that may go undetected using a global approach. For this reason, we are investigating a novel algorithm for detection of localized signal loss, in a similar spirit to the RESTORE approach. Given a whole DWI scan, we will compute voxel-wise percentile DWI images of the median and selected lower and upper percentiles to get stable voxel-wise estimations of the means and variability in the whole scan. Using these percentile images, *z*-score maps can be estimated and large clusters of high *z*-scores will be flagged as localized signal loss artifacts. The removal stage is planned to be the same to the global approach discussed above.
2. Model estimation using biased data: Recently, a method has been published (Gallichan et al., 2010) to correct scans affected by vibration artifacts by explicitly adding a co-regressor term to the model estimation. This method assumes that the vibration artifact is predominantly affecting the left-right direction based on empirical evidence. Based on this assumption, the method first estimates a diffusion model without using data from gradient directions with a strong left-right component. This “temporary” diffusion model is used as a co-regressor to account for the left-right bias when the full diffusion model is computed. The method described by Gallichan et al. uses a manual segmentation of the artifact; we expect that we may be able to use the local detection scheme discussed above to replace this step. Note that this method is aiming at a quick work-around as admitted by the authors, and therefore its validity in the general case needs to be assessed.

As for the simulation-based bias analysis, we are currently working both to integrate this functionality into the DTIPrep framework and to expand it to subject-specific bias analysis. This is a crucial and often overlooked concern: while all scans in a study are usually acquired using the same gradient sampling scheme indicated by the acquisition protocol, the individual scans end up with a modified configuration of gradient sampling after QC. For example, motion correction requires the registration of images along the individual gradient directions to the baseline; the gradient direction in the resulting volume needs to be rotated according to the same rigid transformation. Similarly, the exclusion of a subset of gradients due to various artifacts detected by automatic or visual QC also changes the gradient distribution on a scan-specific basis. A different gradient distribution means a different degree of uniformity and therefore a different amount of expected bias in the diffusion properties estimated from the scan. For this reason, we are expanding DTIPrep to allow users to perform scan-specific bias analysis using the Monte Carlo simulations discussed above.

Finally, it should be noted that dMRI issues are by no means limited to the dominant mode of dMRI using tensor as a model of diffusion, i.e., diffusion tensor imaging (DTI), but rather, affect all dMRI analysis modalities. In fact, it can be argued that analysis via higher-order diffusion models such as Orientation Distribution Functions (ODF's) are more strongly affected by these artifacts, since they require more model parameters to be estimated (Tuch, 2004; Hess et al., 2006). This is because the six-parameter tensor representation, despite its many shortcomings (e.g., inability to resolve fiber crossings, etc.), guarantees considerable redundancy due to the acquisition of many more than the necessary six gradient-weighted images in modern acquisition protocols. In comparison, higher-order diffusion models have a worse ratio of number of parameters to acquired gradient-weighted images. While we focused mainly on DTI examples in this manuscript, it should be noted that the same principles apply to higher-order representations.

### 4.1. Human research and animal research

The results presented here included data acquired from an Autism Center of Excellence network study funded by the National Institutes of Health. Informally called the Infant Brain Imaging Study (IBIS), the network includes four clinical data collection sites, a data coordinating center, and two image processing sites (Hazlett et al., 2012; Wolff et al., 2012). Written informed consent was obtained from parents or legal guardians before enrollment of all subjects, and the study procedures were approved by institutional review boards at each site.

### 4.2. Data sharing

The DTI QC tool presented in this manuscript, DTIPrep, can be downloaded as open source software (Berkeley license) from the DTIPrep project page at the Neuroimaging Informatics Tools and Resources Clearinghouse (NITRC) website http://www.nitrc.org/projects/dtiprep/. The NITRC project page includes manuals, publications, example datasets, bug and feature trackers.

### Conflict of interest statement

The authors declare that the research was conducted in the absence of any commercial or financial relationships that could be construed as a potential conflict of interest.

## Footnotes

^1^http://www.nitrc.org/projects/tortoise/.

^2^http://www.nitrc.org/projects/dtiprep.

^3^3D Slicer (www.slicer.org) is a multi-platform, free and open source software package for visualization and medical image computing.
